# An Inquiry, Critical and Experimental, into the Pathology of Fever

**Published:** 1853-11

**Authors:** N. S. Davis

**Affiliations:** Professor of Pathology, Practice of Medicine, and Clinical Medicine, in Rush Medical College and one of the Physicians to the Ill. Gen. Hospital


*An Inquiry, Critical and Experimental, into the Pathology of Fever* By N S. DAVIS M. D. Professor of Pathology, Practice of Medicine, and Clinical Medicine, in Rush Medical College and one of the Physicians to the Ill. Gen. Hospital.

[CONTINUED FROM PAGE 400.]

On the contrary, if you bring one thousand persons, who have never had continued fever, in contact with a fever patient, under ordinary circumstances, and continue the exposure only the same length of time as in small pox, not an average of five per cent. of the whole number will take the disease; and if the place of exposure is cleanly and well ventilated, the probabilities are that not one of the whole number will sicken from such exposure.

For three winters in succession I have almost daily visited the wards of a hospital, with a class of young men numbering from 50 to 70 persons, in which were received each season every variety of fever, including many cases of the intermittent, remittent, typhoid, and typhus types; some of the latter having been generated on ship-board and accompanied with true typhus and typhoid eruptions during life, and the characteristic post mortem phenomena after death, and yet not an average of two per cent. of these classes have been attacked with continued fever during the whole period of their attendance. Does any one imagine that a similar exemption would have followed the same kind of exposure in wards containing small-pox, without the intervention of vaccination?

Certainly not. For every physician knows full well, that with a similar amount of exposure to Variola, instead of less than two per cent. of attacks, there would scarcely be two per cent. exempt. Hence to say that "the *same kind* of exemption notoriously happens," after exposure to small-pox as occurs after contact with typhoid fever, is an abuse of language. It may be technically true, that one exemption out of a thousand cases exposed, is the *same* in *kind* with ninety-nine exemptions out of every hundred; but practically, in view of important pathological deductions, they are widely different, and point clearly to equally diverse conclusions.

In view of the foregoing considerations, I must repeat the declaration that no definite or useful classification of fevers can be based on their causes.

Reel: 78 - 10 - 5 cont.

Since Morbid Anatomy has become a prominent subject study in the profession, there has been manifested a strong tendency to arrange or classify all diseases according to their supposed seat or primary location.

And this is undoubtedly the most convenient and proper basis for classifying the phlegmasia and other strictly local affections.—But since the attention of the profession was strongly directed to the morbid anatomy of idiopathic fevers, by Clutterbuck, Broussais, Louis, Andral, &c., the same basis has been adopted by many for classifying and diagnosing fevers. Thus Broussais attributed all fevers to simple gastro-enteric inflammation or irritation. Louis, after extending his investigations concerning the morbid anatomy of fevers to the most minute detail, and rejecting the doctrine of Broussais in reference to their local inflammatory origin, nevertheless based his division of continuous fevers almost exclusively on the morbid anatomy of the intestinal canal. In this respect he has been followed by many of the ablest writers of the present day, including Chomel, Gerhard, Jackson, Bartlett, Jenner, and Flint.

So true is this, that M. Louis, in all his later writings, made the presence or absence of lesions in the aggregated glands of the ileum the *test* of diagnosis; so much so, that cases classed as *typhoid during life*, if found, on *post mortem* examination, to be destitute of such lesions, were promptly transferred to the *typhus* class, or set apart as cases of false or *simulated typhoid* disease. The same is true of the more recent reports of Dr. Jenner. In reference to the same subject, Dr. Bartlett uses the following language, viz.: "That the connection between the diagnostic symptomatology of typhoid fever and the entero-mesenteric lesions is, I will not say absolute and invariable, but as nearly so as the connection between the diagnostic symptoms and the characteristic lesions of any given disease whatever in the nosology, in which this connection is not established by positive physical signs."

And in another place the same writer says: "The lesion of the *eliptical plates* seems to me to bear somewhat the same relation to typhoid fever, considered as a disease, as that which their several characteristic eruptions bear to measles, scarlatina, and smallpox." And again, speaking exclusively of typhoid fever as distinguished from typhus, he says: "Other lesions are not accidental, but *essential;* necessary to the disease. *They always enter into its composition.* They make up one of its constituent elements. They are invariably present. This is the case with the alteration of the *eliptical plates* of the small intestine, and the lymphatic glands of the mesentery, corresponding to these altered plates." To make morbid anatomy or disease of a particular structure the basis of a classification of fevers or any other diseases, two things are essential. First, the local structural lesion must invariably be present as an integral part of the disease. Second, such local affection must give rise to the manifestation of a well marked group of symptoms or signs, by which its presence may be clearly recognized during life. For it is evident that local lesions, however constant may be their presence, which can *only* be recognized by a post mortem examination, are of no diagnostic value to the living.

Can we truthfully claim these two essential conditions for the lesions of the eliptical plates or aggregated glands of the ileum and mesentery, so much insisted on by Louis and the modern school of Morbid Anatomists?

Are these lesions invariably present in typhoid fever, and absent in all other fevers? To answer this question satisfactorily, is much more difficult than the casual reader would suppose. For the greater part of this difficulty, however, arises from the fact that there is no agreement among writers as to what shall be called *typhoid fever.* For instance, one class of writers, in their description of fevers, make no distinction between typhoid and typhus. They enumerate symptoms of intestinal lesion, such as liquid stools, tympanitic abdomen, gurgling and tenderness on pressure being made over the iliac regions, &c., as present in some cases and absent in others. In some seasons and epidemics they find these symptoms much more uniformly present than in others. And, as might be expected, they find the same diversity in reference to the presence of disease of the glands of the ileum and mesentery after death. Thus Dr. Thomas Watson, formerly connected with the Middlesex Hospital, London, and a fair representative of the class to which we now allude, says: "Since attention has been drawn to the subject, the patches of glands, and the whole tract of mucous membrane, from the stomach to the rectum, have been diligently explored: and the result seems to be that, at certain times and places, (in other words, in certain epidemics,) the ulceration of the inner surface of the intestines is far less common than at others. It was comparatively rare in an epidemic, of which I witnessed some part, in Edinburgh. Then I came to London; and for several years I never saw a body opened after death by continued fever, without finding ulcers in the bowels. More recently, however, and especially during the present epidemic, (1838) I have looked for them, *carefully*, in many cases that have proved fatal in the Middlesex Hospital, and have discovered neither ulceration nor any other apparent change in the follicles of the intestines."

"The difference,'" continues the same writer, "is very striking between the kind of fever that I witnessed in London, for ten years before the arrival of the spasmodic cholera in this country, and the kind of fever that has since prevailed, and is now (1838) so rife among us. During the first of these periods, there was no eruption to be seen upon the skin; the glands of Peyer, according to my own experience of the fatal cases, were almost invariably affected; and the mortality was very moderate. The present epidemic offers a marked contrast in all these points. A large percentage of those who contract the fever die; after death we seldom detect any disease of the agminate glands of the intestine; the peculiar rash scarcely ever fails to show itself. * * * * You might, I say, almost suppose that I have been speaking of two distinct maladies. But during each of the periods in question, some scattered cases have occurred, bearing most of the characters proper to the other period."

I make the foregoing quotation *in extenso*, because it very fairly represents the views of a large class of British writers, together with some of deserved eminence in our own country. All of this class, of course, regard the local intestinal lesions as very variable; being in some cases present, and in others entirely absent, and consequently in no proper sense, diagnostic of the general disease. Such would necessarily answer the general question under discussion in the negative. On the other hand, a very large class of writers, following the example of M. Louis, restrict their description of typhoid fever to such cases as present evident symptoms of intestinal disease, and reject all others. Thus Dr. Bartlett enumerates among the most diagnostic symptoms, "diarrhœa, the stools thin, watery, and dark or yellowish, sometimes consisting of blood; tympanitic distension of the abdomen, and gurgling upon pressure upon the right iliac region." And Dr. Wood, in his recent valuable work on Practical Medicine, gives so much prominence to these same symptoms that he styles the disease "*Enteric fever.*" To show at a glance how far the diagnosis during life is made to depend on the manifestation of intestinal symptoms, and how closely parallel are all the other phenomena, I will place in opposite columns the symptoms presented during the active stage of the two diseases, taken from the accurate description of Dr. Wood:

TYPHOID.

"The disease being now fairly under way, exhibits the ordinary phenomena of fever; such as frequency of pulse, heat and dryness of skin, flushed face, pains in the head, complete loss of appetite, thirst and great general weakness. The pulse, though sometimes but moderately accelerated, not exceeding 90 or 100 per minute, and of considerable fulness and strength, is in other instances, and especially in females, very frequent, small and compressible, often amounting to from 110 to 120 *or more.*

The flush in the face is of a somewhat more purple tint than in most other cases of fever; and when it is absent, there is not unfrequently a dusky hue of the complexion, with a cer-

TYPHUS.

"The febrile condition, when established, is marked by the usual symptoms of a hot, dry skin, accelerated pulse, hurried breathing, furred tongue, thirst, anorexia and headache. The pulse at this period is often full, and possessed of a certain degree of strength; but it is generally easily compressible, and less firm and tense than in ordinary fevers, with much less appearance of excitement.

The tongue is usually moist and whitish, or yellowish white. Occasionally there is nausea or vomiting; but these are not ordinary symptoms. *The bowels are generally costive, and no stools are procured without medicine.*

Even at this early period the appearance of the face is often tain dulness or heaviness of expression, which may be very slight in some cases but is very striking in others. Headache in some degree is very seldom absent; and not unfrequently it is the chief subject of complaint. The patient often, also, experiences pains in his back or limbs, and sometimes has a feeling of universal soreness, as if bruised or greatly fatigued.

Sometimes there is much restlessness with want of sleep. A characteristic symptom is, in many instances, bleeding from the nose, which, however, is generally slight, and not otherwise important than as a sign. In very many cases, a tendency is observed in the febrile symptoms to remission; sometimes daily, sometimes twice a day.—And occasionally the exacerbations subside with slight perspiration. The pulse becomes more frequent and less strong; the skin acquires a heat and aridity which are often described as acrid or pungent; the obtuseness of countenance and duskiness of complexion deepen; the tongue remains slightly covered, or coats itself with a thicker fur, in either case showing a tendency to dryness or clamminess, and often appearing *red* at the tip and borders; the stomach though often retentive, is sometimes irritable; *diarrhœa is not unfrequent; transient pains are felt in the abdomen, increased by pressure, especially in the right iliac region; and a slight degree of tympanitic distension of the bowels is dis-* peculiar, being of a darkish red, or dusky hue, with injection of the eyes, and sometimes congestion of the mucous membrane of the nostrils and fauces. The mind also exhibits signs of sluggishness, and the thoughts often have some degree of confusion. The headache which is felt most commonly over the brows, is often exceedingly severe, feeling, to use an expression common with the patients, as though the head would burst. These symptoms continue for several days, gradually increasing until the disease attains its height. The surface is now universally hot, with little disposition to perspiration, and the heat is of that peculiar kind, which produces in the hand a sense of pungency as well as burning.

The pulse is remarkably frequent, and generally feeble, beating from 100 to 130, and sometimes as high as 140, 150, or even 160 per minute, so that it can scarcely be counted. A paroxysmal tendency is often observed in the febrile symptoms; an exacerbation generally taking place towards night, and a remission in the morning.

The tongue in this stage usually assumes a brownish color, and becomes more or less dry, especially in the middle, while in some cases a dark sordes begins to collect on the teeth, gums and lips. Sometimes the tongue is clean, smooth and glossy, and sometimes in the progress of the disease it assumes an appearance not unlike that of raw beef. A characteristic eruption now al-

*covered on percussion, with a gurgling sound upon pressure by the hand.*

The urine is sometimes little changed, sometimes scanty, high colored, and offensive. If the surface be carefully examined, red spots like flea-bites will show themselves, usually appearing at first in small numbers upon the abdomen, but afterwards increasing, and sometimes extending to the chest, and even to the limbs and face.

At the same time, inspection will often detect an eruption of small vesicles, called *sudamina*, upon the neck, upper part of the chest, &c."

most always makes its appearance. This consists of numerous small reddish spots, varying in size from a mere speck, to an eighth, a quarter, or even half an inch in diameter, though generally very minute. They are confined to no particular portion of the surface; are generally not elevated, or scarcely elevated, above the surface, and are variously, red, purplish, violet, or almost black.

They are occasionally accompanied with sudamina."

The careful reader cannot fail to be struck with the very close similarity of all the symptoms named in the foregoing columns, except such as depend directly on the condition of the alimentary canal. When they differ at all, it is uniformly in *degree*, and not in kind. So true is this, that Dr. Bartlett himself acknowledges the striking similarity of all the general febrile phenomena, scarcely contending for specific or decided differences in anything except the abdominal symptoms and the *cutaneous eruption*. But the latter is so variable in appearance, and so frequently absent in both the forms of fever, that it can never serve as a basis of diagnosis or classification. In reference to the *rose-colored spots* of typhoid fever, M. Louis reports 90 cases, in 75 of which the spots were observed, and in the remaining 15 they were entirely absent. In the Massachusetts General Hospital, during three years, Dr. Jackson noticed the spots in only two-thirds of the patients.—Chomel and Genest observed them in 54 out of 70 cases. Dr. Wood, of Philadelphia, says: "As the disease has occurred under my own observation, they (the spots) are very seldom absent." On the other hand, Dr. Flint, of Buffalo, in his elaborate Reports on Continued Fever, observes that, "of the whole number of cases in both collections, viz: fifty-five, it (the eruption) was present in thirty-five." And he adds, "careful examinations were made for the eruption in every instance, so that it is hardly possible that it could have been overlooked." Of forty cases of pretty well marked typhoid fever, occurring under my own observation, in the Hospital and private practice, during the past year, the red or rose-colored spots were discovered in only eighteen, and in several of these they were very few and transient.

Not only is the eruption thus inconstant or often absent, but the time or stage of the disease in which it first makes its appearance varies from the second day to the second or third week; differing widely in this respect from those eruptions which characterize the ordinary eruptive fevers, such as measles, variola, &c.

Again the eruptions are not as uniformly distinct and limited, the one kind to typhoid and the other to typhus, as the remarks of some writers would lead us to suppose. The largest and most perfectly developed rose-colored eruption, which came under my observation during the year, was in the case of an Irish emigrant woman, who had been only a few weeks in America, and who presented all the other phenomena of true typhus fever. Dr. Flint gives the following description of the eruption in a case classed by him as of "doubtful origin," viz.: "The eruption is pretty copious over the chest and abdomen, and extends, but sparsely, over the upper extremities. *It is not precisely the eruption of either Typhus or Typhoid, but approaches nearer the latter.* The spots are of a duller red color, are smaller, and not so distinctly elevated, or papular. The redness disappears on pressure, but not in so marked a degree, as is usually the case in typhoid. After repeated examinations, and considerable hesitation, I think the eruption is rather like that of typhoid, than that of typhus" Subsequently he entered upon the record the following, viz.: "*The eruption evidently is of two kinds*, the typhus and typhoid. It extends over the face." On the next day another entry was made as follows: "The eruption to-day presents more the appearance of typhus."

From all this it would appear evident that the cutaneous eruptions in continued fevers are extremely variable, in their constancy, their quantity or number, the time of making their appearance, and their specific character. So true is this, that their presence or absence in any given case, can scarcely be regarded as possessing any diagnostic importance.

If we examine the abdominal symptoms, we shall find them but little less inconstant and variable.

It is true that M. Louis, Chomel, Jenner, and some others, claim the presence of diarrhœa, tympanitic abdomen, and disease of the eliptical plates or aggregated glands of the ileum, in some degree, in all cases of the typhoid, and their absence in those of true typhus.

With these writers, however, no other result could have happened, simply because with them the presence or absence of abdominal symptoms determined the diagnosis. If they were present, it was deemed sufficient evidence to give the case a place in the list of *typhoid* affections. If they were absent, the case was at once placed under the head of typhus. So true was this, that in cases presenting moderate abdominal symptoms during life, with all the other phenomena of typhoid disease, yet if, after death, no changes were found in the mucous membrane and glands of the ilium, they were rejected from the list and assigned a separate rank, under the name of "*Simulated* typhoid fever." With such a mode of diagnosis, it is plain that neither M. Louis nor Chomel, nor their followers in this country, could fail of finding intestinal lesions generally present in the fatal cases of what they called *typhoid* fever. But Jenner went still farther, and in his report on the subject, founded his diagnosis exclusively on the post mortem aprearances, admitting no cases as *typhoid*, that did not present actual morbid changes in the aggregated glands of the small intestine.

A dead-house diagnosis may answer the purposes of the morbid anatomist, but it can scarcely serve any useful pnrpose to the practitioner or his living patients.

On this point Dr. Flint, in his Clinical Reports, makes the following very just remarks, viz.: "Assuredly it is by the ensemble of symptoms during life that the determination of the type of fever is to be made, not to say that in fatal cases more or less importance does not belong to the lesions, either in confirming or correcting the diagnosis. A post mortem test, it is obvious, is too unseasonable for all the practical purposes of diagnosis, and in cases in which recovery takes place, is wholly inapplicable. * *

This, then, is the point which I would enunciate: The determination of the question, whether a particular case of continued fever be of the typhus or typhoid type, should not rest exclusively on the presence or absence of certain intestinal lesions.

It cannot so rest, it is evident, unless the diagnostician has always the advantage of an autopsy.

But irrespective of this, the basis of the division into the two types, is the ante-mortem history. Do the two types possess sufficiently distinctive traits to be nosologically separated? If so, the distinction is undoubtedly in no small measure, strengthened by the fact that certain special lesions are peculiar to one of the types. This is a point to be demonstrably established by examinations after death, of cases in which the typhoid traits were unequivocally declared during life. So also the question whether the lesions peculiar to one of the types are invariably present, is to be settled by repeated examinations after death, of cases in which the distinctive traits of that type were exhibitited during the progress of the disease. The latter question, as well as the former, should thus be fairly and fully met by an appeal to observation. It has been seen that in accordance with this view of the subject, it may probably be proved by reference to Louis' own collection of histories, that typhoid fever may exist, and prove fatal, without disease of the follicles and mesenteric glands. Other cases have been reported."*

In accordance with these views, Dr. Flint has, with great patience and care, recorded the histories of 164 cases of continued fever, occurring in his hospital and private practice, analyzed the symptoms in detail, and attempted a diagnostic classification into typhus and typhoid forms. The result is, that of the 164 cases, 73 are designated unhesitatingly as typhoid, and 65 as typhus, leaving 26 to constitute a list of doubtful cases.

After a very careful examination of the histories of these cases,

* See Clinical Reports on Continued Fever, by A. Flint, M. D. p. 245 6

I find no one symptom present during life in one class, that is not more or less frequently present also in the opposite class. That I may not misrepresent, let the reader examine carefully the following "Summary of Symptoms distinctive of Typhoid and Typhus fever," taken from the 237 page of Dr. Flint's work.

"*Age.*—The maximum and mean age higher in *typhus* than in typhoid.

*Nativity.*—In typhoid, patients are foreign immigrants from different countries, with a certain proportion of citizens of this country. In typhus, they are foreign immigrants, except when the disease is communicated by contagion.

*Season.*—The liability to typhoid *much greater* during the autumnal months; typhus equally and *more* apt to occur during other portions of the year.

*Access.*—Diarrhœe present in a certain proportion of cases of typhoid, not in typhus.

*General Aspect.*—Capillary congestive redness greater in typhus than in typhoid.

*Nervous Symptoms.*—Passive delirium, manifested by incoherent talking, muttering, attempting to get out of bed, &c., present more constantly in typhus; developed *earlier* in the progress of the febrile career in typhus; but very active, persistent delirium, requiring forcible restraint, characteristic of typhoid.

Cephalagia *oftener* present after the fever becomes established, and longer in duration in typhoid.

Vascular injection of *conjunctiva* oftener present in typhus.

Deafness present in a proportion of cases, larger in typhus.

*Digestive System.*—Appetite or relish for food, oftener present in typhus.

A reddened tongue occasionally observed in typhoid, and not in typhus.

Sordes present in a larger proportion of cases of typhus.

Vomiting more apt to occur in typhoid.

Diarrhœa present in one half of the cases of typhoid, and in one third of the cases of typhus; in the latter type always mild or slight, but in the former sometimes prominent as a symptom.

Hemorrhage from the bowels, characteaistic of typhoid.

Tympanites, present in about an equal ratio in both types; but in typhus almost invariably slight, while in typhoid it is often prominent.

Tenderness on pressure over the abdomen, an almost constant symptom in typhoid, and less frequently present in typhus.

Peritonitis from perforation of the intestine, peculiar to typhoid.

*Eruption.*—An eruption present in a larger proportion of cases of typhus; more abundant in typhus, frequently very copious, and extending over the upper extremities, as well as on the trunk; in typhoid seldom copious, often only sparsely scattered over the abdomen and chest, and not present in the extremities. The characters of the eruption are as follows, viz.: In typhoid, rose-colored, oval, slightly elevated, redness momentarily disappearing on pressure. In typhus, of a dull red color, smaller in size, not elevated, redness imperfectly disappearing on pressure. These characters preserved in the two types in the great majority of cases. Occasionally slight variations, and some intermingling of the two kinds of eruption.

*Respiratory System.*—Cough more uniformly present in typhus.

Pneumonits more apt to be developed in typhus.

Epistaxis, extremely rare in typhus, occurring frequently in typhoid. Sputa detached from the posterior nares apt to be tinged with blood in typhoid, not in typhus.

*Circulation.*—Greater frequency of pulse in typhus than in typhoid. The average frequency in the former exceeding an hundred per minute, in the latter falling below that number. Instances of greater frequency occurring oftener in typhus.

Skin, exclusive of eruption and congestive redness, not presenting symptoms distinctive of either type.

Duration, *longer* before death or convalescence, in typhoid, than in typhus; this rule being subject to variations, owing to an unusually short duration of the typhoid type at some periods."

It will be observed that the foregoing summary of *distinctive* symptoms, contains but two items or symptoms that are present in one type and said to be uniformly absent in the other. These are *hemorrhage from the bowels*, and perforation of the intestine, seen occasionally in typhoid, but not in typhus. But, as every one knows, these are purely accidental circumstances, having no *necessary* connection with the general disease, and seen in only a very small proportion of the cases ranked exclusively as typhoid. All the other symptoms are contrasted, not by opposites, or the exhibition of differences in *kind*, but simply by differences in degree. The *terms more* or *less*, *longer* or *shorter*, *slight*, *or well marked*, &c., are those used to designate the differences. And this, too, when nearly one-sixth of the whole number of cases, presenting the greatest intermixture of symptoms, have been separated out to constitute a doubtful list. It may be said that the *eruption* on the skin is an exception to the foregoing remarks; that it is not simply more or less constantly present, and more or less copious, but it is specifically different in the two varieties of fever. But a careful examination of the individual cases given by Dr. Flint proves even this not to be uniformly the case.

I have already quoted his remarks in reference to the eruption in one case where, after repeated daily examinations, he was unable fully to satisfy his own mind as to which variety of the fever it belonged. The difficulty did not arise from the eruption being mixed or made up of two kinds, but its character was so intermediate between the true *rose spots* and the typhus eruption, that it formed a perfect link between the two. I have myself seen three cases during the past season of a similar kind. On turning to the history of the cases classed as of doubtful type, in Dr. Flint's third report, the eruption in case VI. is described as follows, with the appended remarks:—"Presented at the time of his admission an eruption which is described as *dusky*, *not* elevated, and the redness *not* disappearing on pressure. The same characters are recorded the following day. On the next day it is simply noted that the eruption remains. On the next day several *rose spots* were observed, disappearing on pressure."

"*Remarks.*—The variation in the characters of the eruption in this case renders the diagnosis doubtful.

The patient, it will be perceived, did not enter until the sixth day, so that the time of the development of the eruption, and the character which it at first exhibited, are not ascertained. *Aside*

*from the eruption, the symptoms clearly point to typhoid.* The long duration of the access, and the duration of the febrile career; the abdominal symptoms, absence of delirium, and the occurrence of epistaxis, would render the discrimination sufficiently easy, were it not for the *mixed character of the eruption.*"

Case VII., not being lengthy, I quote it in full as follows:—"Charles Madden, Irish, aged 27, admitted in March. He entered hospital three weeks before being attacked with fever, with some trifling ailment, which was not closely investigated. He was able to be up and about until attacked with fever. It is not stated whether he had recently arrived in this country. Access of four days duration. Slight manifestations of delirium on the second night after taking to his bed. Diarrhœa, the dejections being frequent, occurred on the second and third days, but did not afterwards continue. Previously there was constipation. No meteorism nor abdominal tenderness. An eruption appeared on the second day, consisting of three or four *rose spots.* The next day there was a copious eruption, dusky, not disappearing on pressure, and intermingled with a few *rose spots.* Maximum of pulse 114; mean frequency 96. Moderate capillary congestion. Duration of disease 13 days.

*Remarks.*— The *rose spots* and the *diarrhœa, give rise to some doubts as to the diagnosis in this case.* Aside from these, the evidence of *typhus predominates.*

The absence of Tympanites and tenderness over the abdomen; the early development of delirium; the appearance of the eruption early in the disease and its copiousness; the short duration of the disease and the probability of its having been contracted in the hospital, are in *favor* of the *typhus type.*"

From these two cases and a previous quotation, it is certain that Dr. Flint met occasionally with an eruption so compounded of the characters belonging to the *rose spots* of typhoid, and the more papular eruption of typhus that he was compelled to hesitate before assigning it to one or the other; while in one of the cases just quoted, we have *both kinds* of eruption intermingled in what would otherwise be unequivocal *typhoid*; and in the other, the same intermingling, with all the other symptoms of *typhus.*

Nothing could more clearly illustrate the truth of the remark made by Dr. Watson, that "*there is no line of genuine distinction between continued fevers that can be relied on.*"

If, instead of separating one-sixth or one-eighth of the whole number of cases into a list styled *doubtful* or *simulated*, and comparing only the extremes or strongly marked cases; we bring faithfully into the comparison all the cases that can be properly styled *continued fever*, thereby including both the *simulated* cases of M. Louis and the doubtful ones of Dr. Flint, we shall be forced again to adopt the language of Dr. Watson when he declares that "they (continued fevers) run *insensibly* into each other, even the most dissimilar of them."

The reports of Dr. Flint contain a comparatively small number of post mortem examinations, but these are recorded with the same commendable care and faithfulness that characterizes all the other portions of his work, and they embody facts of much interest.

It is well known that the great and all-important pathological fact, claimed to have been established by the *dead-house* investigations of Louis Gerhard, Jackson, &c.; and so thoroughly insisted on by Bartlett, is the development of disease in the *eliptical* plates on Peyer's glands of the small intestines, as *the peculiar* and *characteristic lession* of typhoid fever. "The three reports (of Dr. Flint) contain an account of the appearance of the small intestines in *twenty-two* cases. Of these twenty-two cases, *thirteen* were of the *typhoid* type, eight were of the typhus type, and in one case the patient had lately passed through both types."—The following summary of the appearance is in his own language. "With respect to the typhoid types, in *three* of the thirteen cases, the *Peyerian* patches were notably enlarged, without excavations, and in the remaining *ten* cases the latter, with more or less enlargement existed. Generally, the solitary glands were also the seat of similar excavations, or of great enlargement. The mesenteric glands, also, were invariably increased in size to a much greater extent than in the typhus cases, in some instances being quite large."

"In *each* of the *seven* examinations in cases of this (the typhus) type, the *Peyerian patches* have been somewhat changed, the alteration consisting apparently of a *slight* degree of hypertrophy, causing their development so as to render them visible.—In some instances the patches have been studded with black points, giving rise to what has been termed the *shaven beard* appearance.

Occasionally the solitary glands have exhibited the same kind of development, and the glandular bodies of the mesentery have been *slightly* enlarged, In no case of the typhus type, have the appearances exceeded those just described, but more or less of these appearances have been uniformly observed. Both these facts are important to be borne in mind. *These results prove sufficiently the incorrectness of the statement that the Peyerian patches and mesenteric glands are entirely unaffected in typhus.* So far as these results go, they show, on the contrary, that the Peyerian follicles *are uniformly* affected, but they contain proof only of a certain amount of morbid change, consisting, judged by the gross appearances, simply of a slight degree of abnormal development or hypertrophy."

The question now arises, whether the *slight* affection of the aggregated glands of the intestine, and also of those in the mesentery, described by Dr. Flint as present after *typhus*, is identical with the disease of the same parts after the typhoid, only less in degree? Dr. Flint evidently regards them as different. But on referring to the descriptions of Louis, Bartlett, and others, I find that when death takes place *early* in the progress of the disease, the glandular patches are often found "merely increased in thickness, with redness and softening. Sometimes the *hypertrophy* of the plates and of the subjacent tissue is quite simple, the color and consistence of the membrane remaining unaltered."*

*See Bartlett on Fevers, p. 94.

(*Continued in next number.*)

